# The irrationality of human confidence that an ageless existence would be better

**DOI:** 10.1007/s11017-024-09674-2

**Published:** 2024-06-18

**Authors:** Susan B. Levin

**Affiliations:** Department of Philosophy, Smith College, Dewey Hall, 4 Neilson Drive, Northampton, MA 01063 USA

**Keywords:** Transhumanism, Radical enhancement, Aging, Agelessness, Immortality, Human flourishing

## Abstract

Transhumanists and their fellow travelers urge humanity to prioritize the development of biotechnologies that would eliminate aging, delivering ‘an endless summer of literally perpetual youth.’ Aspiring not to age instantiates what philosopher Martha Nussbaum calls the yearning for ‘external transcendence,’ or the fundamental surpassing of human bounds due to confidence that life without them would be better. Based on Immanuel Kant’s account of the parameters of human understanding, I argue that engineering agelessness could not be a rational priority for humanity on the level of public policy. This stance is complemented by an argument focused on individual decision-making in liberal-democratic milieus, where no governing conception of the good is presumed and the first-personal level matters greatly. Here, drawing on philosopher and cognitive scientist Laurie Ann Paul’s concept of ‘transformative experience,’ I maintain that individuals could not ‘rationally,’ meaning, here, ‘prudentially,’ say ‘yes’ to agelessness. Absorbing the irrationality of human zeal to eliminate aging, based on assurance that an ageless existence would be better, should spur a redoubled dedication to human flourishing.

## Introduction

Today’s controversy over whether humanity should pursue agelessness rests on opposed answers to the question, ‘Is our mortality, which stems, above all, from the inexorability of aging, a good or a bad thing?’ and the closely related query, ‘Would an ageless existence be better?’ Assured that it would be superior, advocates of humanity’s radical enhancement through biotechnology (i.e., transhumanists) urge the avid pursuit of aging’s defeat. Meanwhile, critics insist that an ageless existence would be inferior to humans’ own.

Neither side’s assessments of mortal and ageless existences are demonstrably correct. Yet, in terms of resource allocation, as well as our stance on humanity’s worth and future, much hangs on the resolution of the deadlock produced by the juxtaposition of these positions. This is so all the more due to increasing zeal for aging’s technological defeat (Sect. "A growing fascination with the notion of technologically produced agelessness"). The present paper contributes to breaking this stalemate through its argument that human beings cannot confidently grasp whether an ageless existence would be better or worse and that, as a result, human dedication to engineering agelessness is not rational.

Before launching my defense of the view that confidence in the preferability of an ageless existence is unwarranted, I underscore what a rejection of aiming for agelessness cannot show (Sect. "Death as a “blessing”?"). Here, I focus on the assurance of Hans Jonas and Leon Kass that human mortality is a ‘blessing’ and that agelessness would be worse. Though I agree that humanity should not pursue agelessness, I submit that their confidence in these evaluations cannot be substantiated via argument.

What *can* is the claim that pursuing agelessness, in confidence that the resulting existence would be superior, is not rational for human beings. This is so both on the level of public policy and on that of individual choices to become ageless. Here, ‘rational’ is meant in two distinct ways, one corresponding to each level.

To warrant a commitment of already-scarce, public resources to the technological quest for agelessness, one should have a rational basis for claiming that the resulting scenario would be better. Claims about societal impacts, for instance, on generational relationships and productivity, are necessarily speculative because “dangers and benefits are an empirical matter of which we can have no serious ken” [1, p. 10]. In any case, the more fundamental question is whether there are strong grounds for optimism that *existing* as an ageless being would be preferable. Producing them necessitates an ability to get one’s mind around the idea of conducting an ageless existence. Drawing on Immanuel Kant’s *Critique of Pure Reason*, and more briefly on his practical philosophy, I argue (Sect. "One cannot be confident that an ageless existence would be better: The relevance of Kant") that humanity’s embrace of agelessness as a priority of public policy would be irrational.

In Sect. "Choosing agelessness as a “transformative experience,"" my focus shifts to the plane of individual decision in a liberal-democratic milieu, where first-personal considerations matter greatly; here, ‘rational’ means ‘prudential,’ or ‘in one’s interest.’ Individual readers will be asked to imagine that pertinent biotechnologies are available and that they are weighing whether to say ‘yes’ to agelessness. Though these technologies have yet to exist, considering the individual level now matters because, recognizing the need for public support as a spur to public funding, transhumanists dangle the prospect of agelessness before their readers. In defending the view that saying ‘yes’ would not be rational, I draw on philosopher and cognitive scientist Laurie Ann Paul’s (2014) concept of “transformative experience” [2].

The paper concludes (Sect. "Committing to “internal transcendence”") with a call to reject the yearning for what philosopher Martha Nussbaum terms “external transcendence” [3], instantiated here by the quest for agelessness, in favor of a wholehearted dedication to human flourishing. To proceed otherwise is irrational and greatly impedes that flourishing.

Before proceeding, I offer two clarifications. First, although, technically, the defeat of biological aging would not make one immortal (e.g., accidents could still occur), aging’s defeat would remove the route to death that most preoccupies human beings and the sole, thus far inevitable, hindrance to an unlimited lifespan. Therefore, with this caveat, and because ‘immortal’ and ‘ageless’ are often used interchangeably in discussions of this topic, I do the same. Second, the practical feasibility of aging’s defeat does not concern me here: if biological aging could be conquered, this would not settle the question of whether agelessness should be embraced because science, being but one salient “department of human thought” [4, p. 9], cannot justifiably dictate what humanity should give top priority to.

## A growing fascination with the notion of technologically produced agelessness

A driving interest in the technological delivery of agelessness is evident in the writings of many transhumanists, who represent the exuberant end of the spectrum in today’s debate over the desirability of human ‘enhancement’ through biotechnology.^1^ Within this debate, ‘enhancements/bioenhancements’ are construed as biological manipulations that elevate function or capacity, including rational/cognitive ability and lifespan, absent prior deficiencies as traditionally construed in the realms of health and medicine. Many engaged in this debate reject bioenhancement altogether, while others support it if augmentations do not move their possessors beyond recognizably human levels. Transhumanists themselves dismiss the treatment-enhancement distinction: whatever improves existence—from contact lenses and education to genetic manipulation that radically augmented a select capacity or function—is an “enhancement” [5, pp. 1, 3]. Means of improvement, in and of themselves, are morally neutral [6, p. 522]; the ones to favor in each instance are those that elevate the targeted capacity the most, in quantitative terms.

Transhumanists who urge the conquest of aging are confident that its defeat is not only feasible but the objectively correct focus of humanity’s efforts to handle aging. Disagreement represents a “pro-aging flight from reason” [7];^2^ this claim depends on a conceptualization of aging itself as a disease,^3^ indeed, “humanity’s foremost remaining scourge” [12, p. 659]. Though construing aging as a disease seems like a departure from transhumanists’ rejection of the treatment-enhancement distinction, it is not. For “the right to carry on living” is presented as both a necessary extension of the right to life and as synonymous with “a right to enhancement” [12, p. 662].

Reference to three broad commitments of transhumanists, besides their rejection of the treatment-enhancement distinction, will contextualize further their advocacy of aging’s defeat. First, transhumanists’ urging of aging’s erasure reflects their general view that humanity’s rational and best path forward should be driven by the fundamental biological improvements that (in their view) science and technology will enable in relation to their featured capacities and functions [7, 12, 13].^4^ Second, transhumanists’ pressure for aging’s defeat illustrates their reliance on biological manipulation to address major human challenges—whether they be economic, health-related, focused on moral attitudes, or tied to rights. Third, viewing the erasure of biological limits on lifespan as the rational way to handle aging illustrates transhumanists’ typical stance that more of a capacity that they prioritize is always better [15], with maximization being best [14].^5^

Because transhumanists wish to see their priorities reflected in the allocation of public funds, it is worth noting that, in both the United States and Europe, the quest for agelessness helps to drive transhumanists’ political engagement. Notably, Zoltan Istvan ran for US president in 2016 “on the Transhumanist Party ticket, promising to end death as he drove across the country in the ‘immortality bus’ designed to look like a coffin” [17]. Political parties’ devotion to transhumanism is not always evident from their names; thus, Spain’s Alianza Futurista [18] characterizes itself as a transhumanist entity, dedicated, among other things, to radical life extension (*la longevidad radical*).

Moreover, aging’s defeat is the transhumanist idea that most grips Silicon Valley, with heavy involvement of the ultra-wealthy; for instance, Larry Page (cofounder of Google) and Jeff Bezos (Amazon’s founder) are associated with Calico Labs and Altos Labs, respectively. In Europe, Apollo Health Ventures, based in Berlin, has invested substantially in the conquest of aging, as have several Swiss companies [19]. Also, last year, in the UK, Altos Labs launched the Cambridge Institute of Science [20].

Although, today, transhumanists and their fellow travelers are distinctive for their express pursuit of humanity’s outright transcendence of species-level parameters, important beliefs and attitudes on which they rely with respect to aging reflect a wider cultural milieu, where a strong antipathy to aging and an embrace of its technological defeat are increasingly prominent [21].^6^ Relevant factors, including a repudiation of humans’ “ontological” vulnerability [22, pp. 29, 32] and an intensifying, distinctive perfectionism, provide a tacit backdrop for choices of what to favor or depreciate; this makes the influence of these factors more insidious and potent than it might otherwise be.

To be clear, the embrace of agelessness is not a universal feature of transhumanism, which is an increasingly diverse movement. Alcibiades Malapi-Nelson exempts the quest for agelessness from his endorsement of transhumanism, worrying that “once people…become healthier longer (or stop aging), the creation of new life via offspring may become an after‑thought” [23, p. 391]. For his part, Steve Fuller views “[c]ontemporary transhumanism” as “largely oblivious to the intergenerational consequences of its quest for indefinite longevity” [24, p. 184]. These include the fact that, if aging and death are conquered, then “[r]uptures in the history of human thought and action,” which have “often required new generations unburdened with the experience of their elders,” might fall by the wayside [24, p. 40]. Accordingly: “Who needs to transfer one’s aspirations, let alone genes, to offspring—since, if at first one does not succeed, one in principle has all of eternity to try, try again oneself?” [24, p. 188]. Beyond critiquing transhumanists’ quest for agelessness on a utilitarian basis, Fuller problematizes an ageless existence on grounds of individual self-interest. For example, if “we come to live longer, healthier lives—perhaps indefinitely—we may be simply left with too much time on our hands, such that what now may look like a life of endless leisure turns out in practice to provide endless opportunities for reputational damage through mishaps and misjudgments” [24, p. 167].

Thus contextualized, the present paper can be seen to have a twofold purpose. First, it adds a salient dimension to existing critiques of transhumanism by those who reject it outright, often directing their energies to advocates’ lauding of immortality. Second, since the zeal for immortality is also a point of contention within transhumanism itself, my paper strengthens the position of those transhumanists who reject others’ avid endorsement of agelessness. By doing these things together, my paper defends a position around which critics and some transhumanists can consciously unite; such places have, thus far, been few and far between.^7^

## Death as a “blessing”?

Though their assessments are diametrically opposed to those of transhumanists, Hans Jonas and Leon Kass, too, take it for granted that mortal and immortal existences can be evaluated and compared sufficiently to warrant strong, clear conclusions. Critics of the yearning and quest to eliminate aging, Jonas [25] and Kass [26] contend that what Jonas [25, p. 34] terms “ageless immortality” would be inferior to mortal existence and that human mortality is a “blessing.” This stance is presented as objectively correct: “[A]n immortal…will be less well off than we mortals are now, thanks…to our mortality” [26, p. 6], and “[T]he finitude of human life is a blessing for every human individual, whether he knows it or not” [26, p. 5].

From the likelihood of undesirable results for humanity in general (e.g., overpopulation) if large numbers of people opted for agelessness, it does not follow straightaway that eliminating aging would not be a boon for individuals [25, p. 39]. Thus, Jonas argues separately that

[t]he simple truth of our finiteness is that we could, by whatever means, go on interminably only at the price of either *losing* the past and therewith our real identity, or living *only* in the past and therefore without a real present. We cannot seriously wish either.…[T]he knowledge that we are here but briefly and a nonnegotiable limit is set to our expected time may even be necessary as the incentive to number our days and make them count [25, p. 40].

Kass, too, distinguishes between negative societal impacts [27, pp. 302–305] and effects on individuals: far from being meaningful and purposive, an existence without aging and death would, inevitably, be boring (pp. 308–309). While Jonas [25, p. 40] introduces what can be read as a slight proviso regarding the tie between finitude and meaning (“may even be necessary…”), Kass does not: “To number our days is the condition for making them count” [26, p. 7].

I agree with Jonas and Kass that one should not pursue agelessness—but not because the fact that humans age and die is, incontrovertibly, a ‘blessing.’ The main problem with assertions that humans’ mortality warrants an encomium is humans’ inability to support, via rational argument, the status of this positive assessment as a truth in its own right. For humans are situated, invested interpreters, evaluators, and choosers. What matters here is that, unlike death qua event, finitude—in particular, our awareness thereof (whether or not it is fully conscious)—is woven through human existence;^8^ for instance, it influences the selection and pursuit of priorities requiring dedication and prompts individuals to prize certain interpersonal ties because they recognize that, in the ‘big’ picture, these connections are fleeting. Mortality and concern relating to it are also wrapped up with psychological features like an aspiration for a sense of completeness—a telos that, as Plato suggests in the *Symposium* [30, 189d–193d, 202c–d], seems to lie just outside human beings’ grasp (Kass, too, makes this observation [26, pp. 8–9] but uses it to support his view that even an indefinitely extended lifespan would fail to deliver that sense).

My departure from Jonas and Kass on this issue is subtle, especially because, on balance, they, unlike transhumanists, appreciate the complex nature of human existence. My point is simply that human mortality is not good (or bad) in a free-standing way, as their putting mortality and immortality up for evaluation, both separately and head-to-head, can to be taken to suggest. Not coincidentally, proceeding thus seems most plausible when death is interpreted as an event that happens to (virtually) all living things.^9^

Fortunately, making the case against humanity’s avid pursuit of agelessness does not necessitate one’s ability to offer rationally defensible assessments of mortal and immortal existences themselves. I, too, oppose the pursuit of agelessness, by expressly targeting advocacy of it as the ‘rational’ path for humanity to feature where aging is concerned.

## One cannot be confident that an ageless existence would be better: The relevance of Kant

Based on Kant’s account of human understanding in the *Critique of Pure Reason*, an ageless existence is unintelligible to human beings; this incomprehensibility denies them cognitive access to the object of comparison with their mortal existence, on the basis of which agelessness might be endorsed, with rational confidence, as better.^10^ Moreover, one may helpfully juxtapose Kant’s orientation to death in his practical philosophy against transhumanists’ notion that agelessness is required for flourishing. All in all, appeal to Kant supports the view that the pursuit of agelessness could not be a rational priority of public policy.

### What human understanding can and cannot access

Per the *Critique of Pure Reason*, human beings can cognize only objects of their actual or possible experience [32, A229–232/B282–284, A235–236/B294–295, A677/B705]. Even here, human thought does not reach to things as they are in themselves (*noumena*), disclosing only how they appear (*phaenomena*). This is because space, time, and the categories (e.g., substance, causality) “are not derived from nature and do not follow it as their pattern” (B163). On the contrary, human beings “can extract [them]…from experience only because we have put them into experience” (A196/B241).

Space and time have broader scope than the categories, insofar as categories can have purchase only in spatial and temporal terms. In addition, human beings can understand spatial and temporal existence only in a finite way. This means, among other things, that they cannot rationally grasp something’s existing with a starting point but no termination. Further, according to Kant, the same cognitive frame that enables one to reach rationally warranted conclusions about the external world mediates access to oneself and what is possible for oneself. In the case at hand, what matters is that human beings cannot conceive of themselves in anything other than temporally finite terms [32, B152–159, 275–276].

When undistorted by factors such as false beliefs, human thought about the world and oneself takes a standardized form. In this way, it is objective [32, A89–90/B121–122, B141–142]. But since “experience depends on the standpoint and capacities of the observer,” human knowledge “has a structure of its own, and its own limits too” [33]. Insofar as human cognition is but one kind of rational functioning, it is subjective [32, A297/B353, A306/B362–363, A484/B512, A580/B608, A676–677/B704–705].

Kant differentiates “ideas/pure concepts of reason” from categories: the former are “still more remote from objective reality…for no appearance can be found in which they may be represented *in concreto*” [32, A567/B595, A327/B383]. Nonetheless, humans are continually tempted to stretch reason’s grip to cover ideas/pure concepts of reason, which include the idea of a transcendent being, or God (A235–236/B294–295, A297–298/B354–355, A642/B670).

Transhumanists instantiate this overreaching when they assert that an existence with aging removed would be superior, offering “an endless summer of literally perpetual youth” [34, p. 335]. Transhumanist imagery, such as this, is replete with “hidden promises for happiness, contentment, satisfaction, achievement, and fulfillment” [35, p. 587]. Those who employ it take it for granted that they and their audience have a sufficient grasp of an ageless existence to deem it preferable.

Given Kant’s account in the *Critique of Pure Reason*, the only legitimate way for humans to reference the notion of an ageless existence would be as a *noumenon*, “negatively” construed [32, B307]: as representing an idea that one cannot actually get one’s mind around. This stance is rooted in Kant’s articulation of the view that one cannot conceive of oneself in anything other than temporally finite terms:

Now since for the **cognition** of ourselves, in addition to the action of thinking that brings the manifold of every possible intuition to the unity of apperception, a determinate sort of intuition, through which this manifold is given, is also required, my own existence is not indeed appearance (let alone mere illusion), but the determination of my existence can only occur in correspondence with the form of inner sense, according to the particular way in which the manifold that I combine is given in inner intuition (B157–158).^11^

As the *Critique*’s Schematism of the Pure Concepts of the Understanding makes clear, “the form of inner sense” is time: “The concept of the understanding contains pure synthetic unity of the manifold in general. Time, as the formal condition of the manifold of inner sense, thus of the connection of all representations, contains an *a priori* manifold in pure intuition” [32, A138/B177]. In the case of human understanding and experience, “time-determination is…**universal** and rests on a rule *a priori*” (A138/B177–178).

Time’s identity as the form of inner sense—on the basis of which “all representations,” whether external or internal, can be linked [32, A138/B177]—is evident, as well, in the *Critique*’s Second Analogy of Experience. Here, Kant invokes his stance that “the manifold of appearances is always successively generated in the mind” (A190/B235) to support the legitimacy of humans’ use of the concept, or category, of cause (A191–194/B236–239). What matters here is the Second Analogy’s reiteration of his larger point about time: for appearances to be intelligible to human beings—to be experienced as appearances *of* something or someone—the mind must place them in a temporal sequence.^12^

Apropos of one’s ability to contemplate what an ageless existence would be like, the key point is that, because the form of inner sense is time, humans’ apprehension of their existences depends on it. Given this reliance, the only legitimate way for humans to reference the notion of an ageless existence would be in a cautionary vein, that is to say, as a negative *noumenon*. This way of referencing it reflects Kant’s encompassing view of “[t]he concept of a *noumenon* [as]…merely a **boundary concept**” that helps one to differentiate between what is and what is not accessible to one’s reason [32, A255/B310–311].

When transhumanists, along with Jonas and Kass, reach definite, comparative valuations of mortal and post-aging existences, they presume humans’ ability to grasp what being ageless would involve. In so doing, they treat a post-aging existence as one of Kant’s “positive” *noumena*: an idea beyond one’s comprehension that reason self-deceivingly presumes it can entertain [32, B307–308]. As Paul Guyer observes, per the *Critique of Pure Reason*, “To think that we are entitled to use the concept of a *noumenon* in a positive rather than merely negative sense…is the general form of all metaphysical illusion” [36, pp. 148–149]. A post-aging existence *could* be preferable to humans’ own (this claim has some relation to Kant’s concept of “logical” possibility [32, A220/B267–268, A244/B302]). But what an existence without aging would be like for those conducting it, and hence whether it would be superior to humanity’s mortal one, is unknowable by human beings. From Kant’s standpoint, transhumanists, including Nick Bostrom, Natasha Vita-More, Zoltan Istvan, Ray Kurzweil, and Aubrey de Grey, who urge humanity’s dedication to aging’s defeat on a purportedly rational basis, have succumbed to metaphysical illusion.

As will soon be shown, unfillable gaps and unwarranted extrapolations in transhumanists’ arguments for one’s embrace of their agenda are papered over by their substantial dependence on negation/binaries (e.g., mortal-immortal, pain-pleasure) and analogy. Given transhumanists’ argumentative goals, considerable reliance on such tools is both unavoidable and disallowed. Brief consideration of pertinent material in the *Critique of Pure Reason* will provide a backdrop for my illustration of transhumanists’ overreliance on them.

Transhumanists overestimate humans’ rational access to the referents of negations, or reversals, of parameters of human existence that they wish would disappear. In so doing, they assume that reason can bracket features that, on Kant’s account, are built into human cognition, including the fact that what is accessible to human beings has a finite duration. The negation/reversal of this, namely, ‘infinite’ (or ‘subject to aging’ versus ‘ageless’), does not itself yield a substantive thought. As Kant puts the point,

[I]t is not yet a genuine cognition if I merely indicate what the intuition of the object **is not**, without being able to say what is then contained in it; for then I have not represented the possibility of an object for my pure concept of the understanding at all…but could only say that ours is not valid for it [32, B149].

Human beings cannot cognize anything outside of time and space. In the case at hand, the construction of duration as finite is a fixed parameter of human reason. From a rational standpoint, any other construction of time is merely speculative. Thus, an existence of ‘infinite’ duration is unthinkable by us.^13^

It is important to distinguish between broad and specific levels on which one can assess Kant’s handling of space and time. The broad plane reflects his anchoring of metaphysics in epistemology in the *Critique of Pure Reason*, according to which, per Robert Paul Wolff, human “representation determines the object in the sense that *only through it is the object knowable*.…The realms of being and knowledge are coterminous, and even more significantly, the latter defines the former” [37, pp. 97–98; italics in original]. Thus,

We may abstract from the perceptual peculiarities of the individual, from the particular state of scientific advance of his age, and even from the most pervasive social and cultural biases, but we can never abstract from the subject *qua* knowing human being. If there are forms of perception and cognition which are inherent in the fact of consciousness itself, then these will constitute the limits of Being, so far as it can be discussed at all [37, pp. 320–321].

Such are space and, more pertinently, time. Kant’s specific notion of space, as Euclidean, has been undercut by general relativity, which features four-dimensional spacetime. Kant’s construction of time—that human understanding, of self and other, is necessarily finite and sequential—is not era-specific or comparably contentious. Moreover, one’s rejection of Kant’s three-dimensional view of space, based on subsequent work in physics and mathematics, does not itself jeopardize his broad claim that human understanding depends on one’s contextualization of appearances *in space* [38]. In sum, on the formulation of Ted Humphrey, one “can be aware of something only if it is spatial and temporal and…cannot think away space and time.…[T]hus, any claim that fails to involve essential reference to either space and time or at least time alone must be regarded as unverifiable in principle” [38, pp. 504, 507].

This point, in turn, is key to Kant’s critical investigation of reason, which, as Otfried Höffe observes, “partly legitimises [its] claims…but also, and preeminently, limits them” [39, p. 399]. One need not refrain from attempting to relate oneself to the transcendent, but, in so doing, one cannot depend on reason [14, p. 229]. As Kant puts the point in the preface to the second edition of the *Critique of Pure Reason*, regarding what transcends humans’ own experience, “I had to deny **knowledge** in order to make room for **faith**” [32, Bxxx].

According to Kant, one reason one believes, mistakenly, that one can rationally access the nature of pertinent negations of what *is* thinkable, such as the temporally infinite, is one’s reliance on contrast dependency: “[N]o one can think a negation determinately without grounding it on the opposed affirmation” [32, A575/B603]. This dependence, combined with visceral yearning, encourages humans’ view that they can conceptualize the opposite of a feature or phenomenon that disquiets them, in transhumanists’ case, aging that culminates in death.

Regarding analogy, Kant points out that, when one attributes perfect wisdom and omnipotence to God, one does not thereby “extend our cognition beyond the field of possible experience” but have only “thought this being, which is unknown to us,” by analogy with what is familiar [32, A697–698/B725–726]. As well, predicates like

**very great** or “astonishing”…do not give any determinate concept at all, and really say nothing about what the thing in itself is, but are rather only relative representations, through which the observer…compares the magnitude of the object with himself and his power to grasp it^14^ [32, A628/B656]; [40, 5:139].

In part, transhumanists’ arguments appear to deliver more than they do because undue weight is placed on negation and analogy, as well as on comparatives (e.g., ‘longer,’ ‘smarter’) that stand as evocative placeholders for superlatives whose referents one cannot comprehend. I illustrate this over-dependence through discussion of arguments by Bostrom that include a reliance on negation/binaries, specifically, aging-ageless, pain-pleasure, and dread-bliss, when he projects the nature of existence in a ‘posthuman’ utopia.^15^

Per Bostrom in “Letter from Utopia,” because humans age and die—aka the human body is a “deathtrap” [13, p. 3]—human life is replete with suffering, a terribly “gruesome knot” [13, p. 5]. In contrast, posthumans would be ageless and “built for lasting bliss” [13, pp. 3, 2]; indeed, they would “love life…every instant,” and their pleasure would be boundless [13, pp. 4, 7]. Though, on some level, one can take in these words, one’s thought, imagination, and experience can give no substance to them. As claims about what an ageless existence would be like, they are rationally ungrounded.

Bostrom also relies on analogy, specifically, that between a transition from human to posthuman and the maturation of living things. On the one hand, posthuman existence would be conducted on a higher plane than the human variety [13]. On the other, being posthuman is a future “phase” of human existence, just like the existence of “the flower that follows the seed” [13, p. 1]. On the latter basis, one should identify posthumanity with the consummate actualization of human potential.

Literary devices like metaphor and analogy operate “only against a backdrop of difference” [14, p. 192]. Bostrom’s analogy involving flowers hinges on what is at most a loose parallel, omitting key differences: flowers emerge directly from seeds of their own type (e.g., tulips from tulip seeds), external conditions permitting; meanwhile, posthumans would, in effect, be members of a higher species, engineered through dramatic, biological manipulation. Two very different processes are at work, and only the former involves the actualization and internally steered development of capacities built into an entity as the type of being it is.

The problematic nature of Bostrom’s reliance on an analogy between a human-to-posthuman transition and the maturation of living things becomes even more evident when one considers his direct handling of human ontogeny, in an essay tellingly entitled “Why I Want to Be a Posthuman When I Grow Up” [43]. Here, without express justification, he uses the phrase “human transformation” to cover both intra-human and human-to-posthuman shifts [43, p. 125]. The resulting ambiguity is compounded when Bostrom not only employs but italicizes “radical”—applying it to human ontogeny when his actual target of argumentative persuasion is posthumanity: “Consider…a familiar case of *radical* human transformation: maturation” [43, p. 125]. Because Bostrom’s ontogenetic analogy allows him to capitalize on crucial ambiguities in the key terms “radical” and “maturation,” it plays a far from trivial role in his argument that humanity should embrace the pursuit of posthumanity.

All in all, this ontogenetic analogy is overburdened and sends mixed signals: on the one hand, Bostrom uses it to advocate for human transformation to a higher type of being; on the other, the analogy is supposed to make one more comfortable with the notion of a transformation to posthuman status by likening it to processes that are part and parcel of humans’ own biological, psychological, and social development. But *this* development and the associated life cycle centrally include aging—a perspective in keeping with a (non-transhumanist!) definition of “perfection” in the Oxford English Dictionary as “[t]he most complete or perfect stage of growth or development of a person or thing; maturity; ripeness. Also of a flower: full bloom” [44]. In this context, “maturity,” “ripeness,” and “full bloom” represent transitory pinnacles in relation to entities’ own capacities and functionality—humans, fruit, or flowers, as the case may be. Thus, beyond masking the epistemological inaccessibility of an ageless existence and our resulting inability to confidently evaluate that existence, Bostrom’s ontogenetic analogy assumes that mortality is a difference between human and posthuman existences that is not substantial enough to problematize the analogy’s very use.

The difficulty here is not that an analogy is used but that it is tasked with a role in argument that analogy, as a tool, cannot serve: analogies can function, in a limited way, as heuristics but cannot replace direct argument for the positions that they reflect. What’s more, in the case at hand, direct argument is unavailable. The same applies, in turn, to a reliance on comparatives when superlatives are actually meant but are unfathomable to human beings, as when Bostrom follows the statement “The path to maturity of the soul takes longer” than “seven decades” [13, p. 3] with conceptual reliance on the superlative: “Any death prior to the heat death of the universe is premature if your life is good” [13, p. 3]. The former, comparative statement is comprehensible (e.g., one can conceptualize an average life expectancy of ninety years). In contrast, the latter idea does not correspond to any thought that we can give substance to; *a fortiori*, the associated stance cannot be defended via rational argument.

As illustrated by reference to Bostrom, transhumanists’ argumentative dependence on the above devices papers over the fact that, if one says ‘yes’ to pursuing agelessness, one must be willing to part with one’s humanity, both what humans disvalue and what they cherish [cf. 3, pp. 371–382]. In terms of one’s commitments (professional, interpersonal, social, and so on), one has no sound basis for thinking that, experientially, becoming ageless would be like flipping an aging ‘switch’ to the ‘off’ position, insofar as these commitments are *wrapped up with* humanity’s ‘ontological’ fragility. As a result, one cannot mentally remove, or imagine away, only the fact that one ages, as though it were a self-contained compartment.

But what if one is willing to part with one’s humanity? Speaking of ancient Greeks’ attitudes toward their gods, Nussbaum [3, p. 371] observes that one cannot legitimately claim that existing as they do would be better but only that it would be different. Whether it would be superior to human existence is outside humans’ ability to fathom. Her observation is thought-provoking in relation to transhumanists’ urging that humanity avidly pursue agelessness.

### A brief foray into Kant’s practical philosophy

Consideration of Kant’s views on death and immortality in his practical philosophy strengthens the case for rejecting an avid quest for agelessness. Also, his view that, as far as one can know, one is mortal fits well with his argument in the *Critique of Pure Reason* that one’s cognition of duration is finite.

According to Jeffrey Church, the importance to Kant’s practical philosophy of the fact that we die well surpasses his express attention to it: death “plays an important, albeit indirect role in framing Kant’s fundamental understanding of the worth and dignity of humanity, achieved through our capacity, unique among animals, to liberate ourselves from our instinct for self-preservation and to pursue the moral law” [45, p. 310]. In short, “Our eventual deaths are the context in which humanity’s moral aspirations take place” [45, p. 318].

Per Thomas Hobbes, the fear of death is foremost among human “Passions” [46, XIII]. As Church sees it, Kant rejects Hobbes’s view that humans, like nonhuman animals, “are motivated at bottom by calculations of pain, pleasure, and, most importantly, our fear of death. Kant was dissatisfied with this lowly conception of the human being, who slavishly attempts to evade death at all costs” [45, p. 310]. Quite simply, per Kant in *Lectures on Ethics*, “The man of inner worth is not afraid of death” [47, 27:376]. Here, transhumanists align with Hobbes, not Kant. Thus, *mutatis mutandis*, de Grey harks broadly back to Hobbes when insisting that—because it makes death inevitable—aging is “humanity’s foremost remaining scourge” and that “the right to carry on living” is built into the right to life [12, pp. 659, 662].

Kant’s emphasis on finitude and rejection of humans’ fear of death are not the whole story because, in the *Critique of Practical Reason*, he endorses the soul’s immortality as a practical “postulate,” or “presupposition,” of morality [40, 5:122]. Since “the moral law gives us justification to have faith that death is not the end,” the postulate of immortality is a second reason that, for Kant, death “is not the *summum malum* Hobbes took it to be” [45, p. 312].

Kant invokes the practical postulate of the soul’s immortality as a condition for completing the moral project, namely, perfecting the will, that each human being should put front and center [40, 5:4, 122–124]. Not only would individuals need more time for this than humans’ biological frame allows, but humans’ creaturely nature is a source of impediments to the moral life [48, Sixth Thesis, 23; 47, 27:543–544]. Rationally unverifiable, immortality is salient as a practical postulate only: what matters is that it “contain no intrinsic impossibility (contradiction)” [40, 5:4].

Transhumanists’ view of flourishing hinges on something far stronger: a known opportunity literally to achieve “an endless summer of…perpetual youth” [34, p. 335]. Moreover—Bostrom’s ontogenetic analogy notwithstanding—immortality would not complete a distinctive project of humans’ own, as is the case for Kant, but would, instead, replace humans’ mode of existence with what is purported to be a more valuable kind.

### The nature and desirability of an ageless existence: Further reflections spurred by Kant

If one is to rationally commend the pursuit of agelessness as a priority of public policy, one should have warranted optimism that an ageless existence would be better. As I have argued, there are two sets of issues here, one involving understanding and experience (Kant’s province of theoretical reason), the other morality and living well (the sphere of practical reason). Further consideration of these strengthens the case for rejecting transhumanists’ confidence in the superiority of an ageless existence.

Grasping existences with and without aging is a precondition for offering rationally defensible comparisons between them. Humans cannot think of existence outside of time or at least outside of time as they experience it. Moreover, of the two “pure forms of sensible intuition” [32, A22/B36], time is more encompassing than space:

[S]ince…all representations, whether or not they have outer things as their object, nevertheless as determinations of the mind themselves belong to the inner state, while this inner state belongs under the formal condition of inner intuition, and thus of time, so time is an *a priori* condition of all appearance in general, and indeed the immediate condition of the inner intuition (of our souls), and thereby also the mediate condition of outer appearances [32, A34/B50].

Due to its encompassing role for humans as the beings they are—and because the effects of agelessness, if any, on the experience of time are unknown—human reason cannot compare existences with and without aging, except in a way that construes an ageless existence as an existence that has a long*er* duration.

Now, suppose that science and technology could deliver biological agelessness through the manipulation of genes and cells. Unless cognitive manipulation occurred at the same time, ageless beings are likely to retain the same cognitive apparatus as unenhanced human beings, central to which is what Simon Gurofsky aptly calls the “always-already-spatio-temporal” character of human knowledge [49, p. 841]. If they did, they would be able to conceptualize their literally infinite existences only in finite terms. Subjectively (meaning, here, individually), however, ageless beings might experience themselves as profoundly changed, in phenomenological ways that their cognitive frame could not accommodate. And, if this occurred, the dissonance between subjective and (humanly) objective standpoints on the experience of being ageless could lead to an immense frustration and profound estrangement that had no human precedent. My remarks in this paragraph are, obviously, speculative: their role is simply to illustrate a not-implausible outcome that would be unwelcome to transhumanists.

Moreover, in his practical philosophy, Kant suggests that experiencing one’s life as long may be a sign of disengagement [47, 27.383]. Here, as elsewhere in *Lectures on Ethics* [47, 27.375–381], he observes that the duration of existence and one’s level of fulfillment need not fit together hand in glove. In other words, at least in the case of humans, a longer existence is not necessarily a better one: if one already prioritizes things that degrade one’s humanity (e.g., appetitive over-indulgence, worldly reputation), then having an indefinitely larger number of experiences involving them does not obviously make life better. Indeed, in all likelihood, it makes it worse [40, 5:118; 47, 27.378–381, 384–385, 664–665].

Further, reference to Kant allows one to differentiate (1) goods that are, at best, instrumental and ends that are proximate from (2) a telos that is ultimate because it fulfills human nature. As Church puts the point, human beings evince “their dignity by surmounting our fear of death,” which keeps humans’ focus on the merely physical and contingent, “and staking our lives for moral ends” [45, p. 310].

Whether one endorses the content of Kant’s view of living well, centered on freely conforming one’s will to the moral law [40, 5.130–132], is irrelevant here. The point is that having more time to exist matters only insofar as it serves a worthy, ultimate telos for human beings—one that, unlike transhumanists, Kant, for one, expressly defends as such.

What comes across, in transhumanists’ writing, is their fervent yearning that aging be obsolete. But this desire, no matter how strong, does not itself warrant plowing ahead: if transhumanists expect their audience to consider seriously their urging of aging’s elimination, they owe their readers a defense of a non-fantastical “that for the sake of which” (*hou heneka*; Aristotle, *Physics* 194a28 [50]) that can be scrutinized and assessed. All too often, transhumanists simply assume that an existence without limits under which they chafe will be blissful and that maximizing capacities that they see as all-purpose goods will produce an exalted existence, without giving credence to the idea that “sheer ability does not have merit independently of the ends it serves” [14, p. 158].

These assumptions are grounded, not in reasoned argument, but in desires that gain their purchase from what, in human existence, displeases transhumanists, together with fantasies of what life without that would be like.^16^ In the terms of Kant’s practical philosophy, they are “inclinations” [40, 5.117–118], which “can *motivate* and *explain* actions…but…are not enough to *justify*” them [33]: only an ultimate end can do that. Thus, on Kant’s account, recognition that, as far as human knowledge extends, humans are mortal and dedication to a guiding “that for the sake of which” go hand in hand.

In relation to Kant, transhumanists’ advocacy of agelessness is not errant merely because it succumbs to metaphysical illusion. It is flawed, as well, from the standpoint of his practical philosophy. For Kant, as we have seen, the existence of God and an immortal soul are practical postulates for the sake of human flourishing. For transhumanists, in contrast, agelessness is an apprehensible idea that corresponds accurately to a kind of existence that will (with suitable effort) come literally to exist. Moreover, by their lights, aging must be conquered if genuine flourishing is to occur: by transhumanists’ ‘objective’ standards, human limits, central among them the brevity of life, preclude the achievement of rich, fulfilling lives; therefore, those who can and will flourish are future, hypothesized posthumans. From a Kantian standpoint, transhumanists’ stance is rationally indefensible, through and through.

Like it or not, human flourishing, or what Nussbaum terms “internal transcendence” [3, pp. 380–382], is the sole type of living well that human beings can conceptualize and direct their attention toward. The impact of transhumanists’ urging that humanity prioritize the pursuit of agelessness will be severe if it diverts human beings from a dearly needed, redoubled dedication to it. Unlike the quest to remove framing parameters of human existence, that for internal transcendence calls on human beings “to bound our aspirations by recalling that there are some very general conditions of human existence that are also necessary conditions for the values that we know, love, and appropriately pursue” [3, p. 379]. It is entirely rational “to want, always, to be better” [3, p. 381]. But, in so striving, one must appreciate that one’s most cherished commitments and ends are propelled by “values in and for [a human] context—which, like all contexts…contains and is structured by limits as well as capabilities” [3, p. 378]. Concerning aspiration, exemplariness, and fulfillment, “apart from the contexts of specific forms of life, we can…say nothing with real content” [3, p. 378]. In his own way, Kant would agree.

With all this inbuilt uncertainty about a post-aging existence and how it might compare with humans’ own, it would be irrational to make agelessness a human priority. This conclusion is reinforced in the following section.

## Choosing agelessness as a “transformative experience”

I now consider the rationality (or not) of embracing agelessness from a different perspective: that of individual decision-making in a liberal-democratic milieu. Here, no governing conception of the good is presumed; the first-personal level matters greatly; and ‘rational’ means ‘prudential.’

Why address this level at all given the argument of the previous section, combined with the fact that age-defeating biotechnologies do not currently exist? Considering the first-personal level now matters because, as transhumanists are well aware [34, p. 335; 51, p. 1], the likelihood that public policy will favor the development of these technologies is greater if strong public interest is expressed, and the result would be the subordination or even elimination of other priorities. Moreover, given the visceral appeal of ‘eternal youth,’ public support could be spurred if many people came to believe that aging’s defeat would be imminent if only humanity invested suitably in the project. Consideration of the first-personal level in this section of the paper problematizes the assumption that biotechnology would deliver an experience of flourishing that, for human beings, is all too elusive. Seeing the great uncertainty of existence following the use of biotechnology that made one ageless and the stakes of signing on should reduce zeal for the human quest to engineer agelessness. In this way, the argument and conclusions of this section reinforce those of Sect. "One cannot be confident that an ageless existence would be better: The relevance of Kant."

Strikingly, beyond claiming that, rationally speaking, aging’s defeat should be the dominant focus of biomedical research [34, p. 22], de Grey can be taken to suggest that individuals’ refusal to *use* age-defeating technologies would be irrational. For, on one interpretation of his expanded right to life [12, p. 662], it is like humans’ right to education, namely, a “primary,” or “all-purpose,” good that “one could not rationally forswear.…Anyone who could wish to waive it must simply be ignorant of what is good for him” [52, p. 121]. Thus, one conducting her existence rationally would not decline biotechnologies that conquered aging. This interpretation of de Grey’s extended right to life is reinforced by his conceptualization of aging itself as humanity’s gravest disease, refusing a cure for which would be supremely irrational.

If saying ‘yes’ to aging’s erasure is the rational course for individuals, then subjective considerations are irrelevant. This stance toward aging’s refusal is starkly at odds with the prominence that first-personal considerations are touted to have, within liberal democracy, in decisions that humans routinely make during the life course. Moreover, within liberal democracy, the salience of these factors is not negotiable; thus, for instance, the ‘harm principle’ has been employed to preclude “wrongful interference in individuals’ lives” based on others’ values and indignation [53, p. 474]. The justification for prominence given to first-personal factors is arguably even stronger if the choice involves whether to change a species-level parameter, as would be the case here.

Where the rationality of individual decisions is concerned, the more appropriate question is, ‘*Could* an individual rationally choose immortality, in confidence that the resulting existence would be better?’ Based on L. A. Paul’s [2] fruitful concept of “transformative experience”—which is only now being brought to bear on transhumanism [54]—the answer is ‘no.’ Paul’s frame is helpful, with a key proviso. On Paul’s account, all transformative choices—from choosing a career to deciding whether to become a vampire—have the same structure, and epistemic shifts differ only by degree [2, pp. 50, 11]. Based on my consideration of Kant, I maintain that Paul’s view gives insufficient weight to differences between situations where—whatever one chooses—one will undoubtedly remain a human being and those in which one’s options include jettisoning key parameters of human existence for the sake of a ‘better’ existence.

According to Paul, transformative choices, which “constitute a central part of a normal life” [2, p. 50], have two main aspects. One is “epistemic”:

When a person has a new and different kind of experience, a kind of experience that teaches her something she could not have learned without having that kind of experience, she has an *epistemic transformation*. Her knowledge of what something is like, and thus her subjective point of view, changes [2, pp. 10–11].

The second facet is “personal”:

The sorts of experiences that *can* change who you are, in the sense of radically changing your point of view…are experiences that are *personally transformative*.…If an experience changes you enough to substantially change your point of view, thus substantially revising your core preferences or revising how you experience being yourself, it is a personally transformative experience [2, p. 16].

Because transformative experiences are two pronged, “You don’t know how many of your core preferences will evolve” [2, p. 81].

According to Paul, if, to qualify as rational, transformative decisions should be based on one’s “assessments of what the experience would be like and what this would imply about the subjective value of [one’s] future lived experience,” an individual “cannot rationally choose” any particular transformative experience [2, pp. 19, 18]. The reason is that,

when we are supposed to choose, we *cannot know* the relevant values, either because our pre-choice self cannot assign them or because they will change in unknown ways.…If you cannot determine the expected value of an act, you cannot have value-based reasons for choosing that act, and so you cannot use these reasons to guide your reasoning about that act in a way that falls within the scope of normatively rational decision-making [2, pp. 33, 34n42].

Crucially, transformative decisions can be rational, if at all, only in an extended sense: one makes a certain choice because she wishes to see how having done so will impact her [2, p. 135]. When one opts to

have a transformative experience, we choose to discover its intrinsic experiential nature, whether that discovery involves joy, fear, peacefulness, happiness, fulfillment, sadness, anxiety, suffering, or pleasure, or some complex mixture thereof.…A life lived rationally…as each big decision is encountered, involves deciding whether or how to make a discovery about who you will become [2, p. 178].

Not only could a complex blend of the above result from one’s choice, but peacefulness, or a lack of disturbance—which sounds strictly positive—could take divergent forms like rich tranquility and apathetic numbness. Since one cannot rationally anticipate what will ensue, even broad optimism about what awaits is unwarranted.

Paul assimilates her vampire scenario to major decisions that one actually makes during the life course, where opportunities may exist to consult helpfully with others who have made a particular choice that one is contemplating: thus, the technology to make one a vampire is available; moreover, humans close to one have already taken the plunge, assuring one that life as a vampire is better [2, p. 43]. It may seem “obvious that you *should*…choose immortality, superhuman powers and intense new sensory experiences—that sounds great!” [2, p. 47]. But, as with career and family decisions, individuals must ask themselves, “What basis do you have for your belief? None…that is epistemically justified” [2, p. 47]. Though Paul notes that—unlike decisions that human beings actually make during the life course—the decision “to become a vampire…would involve a change in species,” she insists that the same “structure of transformative choice is reflected” there [2, p. 50].

When it comes to evaluating the quest never to age, Paul’s frame is helpful, with a key proviso: her view that transformative decisions are structurally identical and that epistemic shifts involved differ only by degree [2, pp. 50, 11] does not adequately capture key distinctions between scenarios that individuals routinely confront during the course of their lives as human beings and those in which one option is to become a purportedly more elevated kind of being.

Based on Paul’s account, one should not assure oneself that existence after a major decision will be any better, let alone dramatically so, even when all options are ensconced within the human realm. Yet there is a great deal one can do, based on one’s own experience and priorities, to reduce uncertainty: make careful lists of pros and cons; converse with others we know who have previously chosen options currently before us; and read published accounts of persons unknown to us who have made pertinent decisions. One might also consult works of fiction, from Greek tragedy to contemporary novels, which can richly influence one’s emotions and intellects in ways that have practical (e.g., moral) bearing [55, pp. 69–92; 3, pp. 365–391; 56, pp. 53–78].

In my view, by building in the option to consult friends who have already said ‘yes’ to becoming a vampire, Paul’s decision scenario here embeds too much of what needs to be shown, that intra-human decisions and those including an option to make a species-level shift are relevantly alike. In any case, the issue in this paper is whether, today, when aging’s defeat is not yet practicable, an individual could rationally, meaning ‘prudentially,’ be confident that her existence without aging would be better. In this instance, helpful options, such as consulting others who have already made the choice, are unavailable.

Bostrom assures the reader that, compared with priorities of those who aged, ageless individuals would have superior “vaults of value” [13, p. 4]; at minimum, once ageless, individuals would be better positioned to pursue what they already cared about [13, pp. 1–2]. Given that one prong of this decision scenario, saying ‘yes’ to agelessness, involves a change in a species-level parameter, Bostrom’s confidence that a non-aging existence would be superior—and, thus, the prudential choice—is unwarranted. As A. G. Gorman observes, “While it may be possible for some people to have meaningful infinite existences, no one has much warrant for believing that she herself is such a person” [57, p. 1081].

It is highly debatable whether the choice to become ageless could be rational even in Paul’s extended sense of taking a flyer. Though, depending on the circumstances, it may be self-destructive or immoral to do so, individuals can live in such a way that detachment from particular commitments, dependencies, and responsibilities is always on the table. But it would be quite another matter for individuals to say ‘yes’ to whatever ensued in relation to these *facets* of human existence, proceeding as though they, and the finitude giving them focus and direction, were inessential.

According to Adam Buben, “Individual constitution…could actually dictate one’s suitability for something indefinite or even infinite” [58, p. 1143].^17^ This is possible, to be sure. But the connection between an individual’s constitution as a mortal being and her suitability for an infinite existence could also be looser than Buben suggests. The choice to become ageless may appeal most to persons inclined to see their lives as self-curated adventures, detachable from extra-individual commitments, dependencies, and responsibilities. In addition, I grant that they may be more likely to number among those for whom the decision panned out well. However, it is not implausible that such individuals would come to regret adherence to the sense of stark self-sufficiency that had spurred their choice to erase aging. I also find it plausible that, conversely, far less adventurous persons who felt stultified by the burden of choice within mortal parameters might experience agelessness as liberating.

The bottom line is that deep uncertainty is built into the nature of this choice. Living well, or flourishing, simply cannot be considered and evaluated in a context-independent way: the only type that one can address with any authority stems from the cultivation and actualization of one’s own, human capacities. This path can give rise to a vast range of specific lives, but, change any anchoring parameter, and one must consider afresh what living well consists in—for the resulting type of creature. Contra Bostrom, there is no good reason to believe that the erasure of aging would make one better able to achieve one’s priorities when mortal or spur significant improvements in what one valued most. Though major shifts might occur, it is an open question whether the resulting existence would be better [15, 59].

## Committing to “internal transcendence”

Contra transhumanist de Grey [7], the last thing that human advocacy of aging’s erasure could be is “dispassionate.” The passion at issue here is for liberation from parameters of human existence experienced as deeply unpleasant and disquieting. In relation to the quest for agelessness, what is distressing is not the event of death per se but what death *represents* for the self-conscious, temporally embedded beings that *humans* are.^18^

As Nussbaum [3] maintains, absorbing that confidence in the superiority of an ageless existence is irrational and taking to heart what death represents should help to spur a redoubled dedication to “internal transcendence”; here, capacities that frame human existence are seen clearly to constitute the setting within which an existence intelligible to human beings can be meaningful and purposive at all. This form of transcendence equally involves surpassing by elevation and “a transcending by *descent*, delving more deeply into oneself and one’s humanity, and becoming deeper and more spacious as a result” [3, p. 379]. It sets an appropriately high bar for flourishing, such that “if one really pursued [it] well and fully…there would be little time left to look about for any other sort” [3, p. 379].

Where aging is concerned, rejecting “external transcendence” [3, pp. 380–382]—the yearning to become, in effect, a different type of creature—can motivate a conscious integration of mortality in a holistic view of human existence, accompanied by recalibration of affective responses to humans’ mortality. One may be led, at minimum, to temper one’s “rage at and hatred of…death, which [one] would still appropriately fear and avoid” [3, p. 379]. This affective modulation stands to improve both one’s self-interpretation and one’s attitude to others, which are always intertwined [63]. Importantly, it might disincline one to “[withdraw] love and concern from that which cannot be stably controlled,” seeing as worthwhile solely what one fantasizes to be “immune from change and alteration” [3, pp. 379–380]. Since the understanding of self and ‘other’ are inseparable, the aforementioned adjustments to motivation stand to ameliorate both a tendency to shun one’s own aging and a disvaluing of older persons for representing what one dearly wishes to stave off in one’s own case [64].

If one embraces transhumanists’ quest for agelessness, one does two things, both of which are deleterious to human flourishing. First, one solidifies and institutionalizes a view of humanity—especially, but far from entirely, ‘older’ persons—as fundamentally inadequate, or defective. This view is built into de Grey’s classification of aging as a disease, humanity’s worst. Lest one think that intensified ageism would be limited to those who were, say, over 65 or 70, it should be noted that, not only is a concern to avoid aging stronger than ever among those in ‘middle age,’ but the phenomenon of “prejuvenation” represents the growing priority of this even for persons in their 20s [65].

Second, if one endorses the quest for agelessness, one telegraphs one’s acceptance of the notion that the prime route to addressing major human challenges is direct manipulation of humans’ biological endowment. Pinning one’s hopes on this course reflects and would likely intensify a subordination of commitments to address challenges (e.g., educational inequities, damage to the natural environment) that require intensive, ongoing dedication and do not admit of technical solutions.^19^

The last thing one should do is join transhumanists and their fellow travelers in casting aspersion on existing as human beings, largely ignoring a panoply of measures (involving education, water quality, the physical environment, and so on) that are known to improve human existence—and are supremely cost effective given their boons—but have yet to receive humanity’s full-scale dedication [14, p. 163]. The seemingly ordinary is where one often finds opportunities to have extraordinary impacts, which might become gloriously familiar if one were to see humanity with less distortion by viscerally alluring, yet false, notions of where fulfillment lies.

## Footnotes

1 I characterize thinkers as transhumanists based on the content of their ideas and aspirations, regardless of whether they apply the label to themselves.

2 Here, de Grey voices a common charge by transhumanists that disagreement with their views is irrational [8, pp. 357, 366; 9; 10, p. 54].

3 Though arguing the point falls outside my purview here, I should note that I find this conceptualization of aging to be implausible; for instance, “[U]nlike any disease, age changes cross virtually every species barrier…occur in all animals that reach a fixed size in adulthood and only after sexual maturation” [11, p. 3].

4 This view has been vigorously contested [14].

5 That maximization of a valued capacity is the optimal course is far from obvious. This is clear already in the case of human intellect [16].

6 This paper draws selectively on Levin [21], which addresses cultural roots of transhumanism where zeal for agelessness is concerned.

7 Since my paper concentrates on the position of those transhumanists who urge humanity’s pursuit of agelessness, hereafter, unless otherwise evident from the context, “transhumanists/transhumanism” refers to that group.

8 Cf. Heidegger’s differentiation of “death” from both “perishing” and “demise” in *Being and Time* [28, §§49–50]; per Haugeland [29, p. 66], while demise, unlike perishing, is relevant solely to Dasein among living things, “neither is to be identified with death, *existentially* conceived.”

9 As to why the proviso “virtually” is needed, see Guest [31] on the “immortal jellyfish.”

10 On the relevance of the *Critique of Pure Reason* to an assessment of transhumanism, see further Levin [14, pp. 221–230].

11 Bold type is Kant’s.

12 As a result, direct assessment of Kant’s defense of the categories themselves in the Transcendental Deduction falls outside the purview of this paper.

13 Unlike Kant, transhumanists focus on the worldly manufacture of what corresponds, in Kant’s terms, to an idea/pure concept of reason, namely, an ageless existence. This difference is irrelevant here, as what matters is one’s inability to give content to the idea.

14 Translation [32] slightly modified.

15 My claims concern the significance of Bostrom’s argumentative reliance on these devices, building in no assumptions about his intentions in relation to it. As well as differing from Kant, in their conviction that reason suffices for envisioning what transcends human beings, transhumanists diverge from Christian thinkers, who usually prize not only rationality but also humility, faith, and adoration [41]. Even for Saint Thomas Aquinas, a strong partisan of reason among Christian theologians, a divine assist via faith and grace is crucial to any reduction of the epistemological gap between us and God (*Summa Theologiae* [42], I 12.1c, 12.4sc, 12.5c).

16 For exploration of desires to surpass humanity, including the yearning for immortality, from a psychoanalytic perspective, see Dauphin and Abell [35].

17 Buben claims that, “[a]ll things considered, the best form of immortality…involve[s]…indefinite life-extension with the possibility to opt out in case existence becomes intolerable,” that is to say, “loses its luster” [58, pp. 1141–1142]. Though the availability of an “eject button” [58, p. 1143] would be preferable to its absence, Buben’s presentation suggests an individualistic construction of meaningfulness and living well that requires further defense. Further, he has a “generally optimistic attitude about the prospects of meaningful immortality” [58, p. 1140]. If this attitude framed humanity’s commitment to immortality, and individuals were less than enamored of an ageless life, their reaction might be viewed as signaling personal failure (e.g., deficient imagination or will). In addition, Buben [58, p. 1138] observes that, for mortal beings, there is no such thing as simply *reversing* course, in order to embrace an option previously considered but not selected: that decision point has come and gone, and what flowed from it is not extractable from one’s identity. I submit that the same relationship could obtain between an immortal individual’s saying ‘no’ to the continuation of an ageless existence and the prior point when she said ‘yes.’

18 See Heidegger’s [28] handling of human mortality and temporality in *Being and Time*, as discussed by Levin [60, pp. 162–165]. Given humans’ focus on what death represents, arguments against the quest for agelessness that emphasize humans’ likeness to practically all other life forms—death’s being a shared, biological reality [61, p. 58]—are incomplete because it is not mortality qua biological phenomenon that ultimately spurs humans’ drive to surmount it [62, pp. 103–106].

19 These commitments accompany not only transhumanist advocacy of the categorical augmentation of their favored capacities as rapidly as possible but also the “weak transhumanism” defended by Brennan [66], who does not dispute transhumanists’ goal of “radical enhancement” but only their view of pacing (p. 245).

## References

[1] Quilter John G (2016). The new enhancement technologies and the place of vulnerability in our lives. Theoretical Medicine and Bioethics.

[2] Paul LA (2014). Transformative experience.

[3] Nussbaum Martha C (1990). Love’s knowledge: Essays on philosophy and literature.

[4] Midgley Mary (2011). The myths we live by.

[5] Levin, Susan B. 2014. Transhumanism and enhancement. John Wiley and Sons. 10.1002/9780470015902.a0024136.

[6] Savulescu Julian, Steinbock Bonnie (2007). Genetic interventions and the ethics of enhancement of human beings. The Oxford handbook of bioethics.

[7] de Grey, Aubrey D. N. J. 2008. Reasons and methods for promoting our duty to extend healthy life indefinitely. *Journal of Evolution and Technology* 18(1). http://jetpress.org/v18/deGrey.htm.

[8] Harris John, Holm Søren (2002). Extending human lifespan and the precautionary paradox. Journal of Medicine and Philosophy.

[9] Bostrom Nick, Ord Toby (2006). The reversal test: Eliminating status quo bias in applied ethics. Ethics.

[10] Savulescu Julian (2013). Making better babies: Pro and con. Monash Bioethics Review.

[11] Hayflick, Leonard. 2003. Has anyone ever died of old age? In *Has anyone ever died of old age?* Leonard Hayflick and Harry R. Moody, 1–4. New York: International Longevity Center.

[12] de Grey ADNJ (2005). Life extension, human rights, and the rational refinement of repugnance. Journal of Medical Ethics.

[13] Bostrom, Nick. 2020. Letter from utopia. Version 3.3. https://www.nickbostrom.com/utopia.pdf.

[14] Levin Susan B (2021). Posthuman bliss? The failed promise of transhumanism.

[15] Hofmann Bjørn (2017). Limits to human enhancement: Nature, disease, therapy or betterment?. BMC Medical Ethics.

[16] Pollet Edith, Schnell Tatjana (2017). Brilliant: But what for? Meaning and subjective well-being in the lives of intellectually gifted and academically high-achieving adults. Journal of Happiness Studies.

[17] Smith, Wesley J. 2020. A transhumanist runs for president. *National Review*, 22 February. https://www.nationalreview.com/2020/02/transhumanism-zoltan-istvan-technological-self-perfection-immortality/. Accessed August 29 2023.

[18] Alianza Futurista. 2023. http://alianzafuturista.org/index.html. Accessed July 2 2023.

[19] Billing, Mimi. 2021. European investors have a new obsession: Longevity. *Sifted*, 21 December. https://sifted.eu/articles/anti-aging-longevity-investors. Accessed August 29 2023.

[20] Scialom, Mike. 2022. Altos Labs launches Cambridge Institute of Science with cellular rejuvenation mission. *Cambridge Independent*, 1 July; updated 3 July. https://www.cambridgeindependent.co.uk/business/altos-labs-launches-cambridge-institute-of-science-with-cell-9261846/. Accessed August 29 2023.

[21] Levin Susan B (2024). The less visible side of transhumanism is dangerously un-radical. Techné: Research in Philosophy and Technology.

[22] Turner Bryan S (2006). Vulnerability and human rights.

[23] Malapi-Nelson Alcibiades (2019). Transhumanism, posthumanism, and the Catholic Church. Forum Philosophicum.

[24] Fuller Steve (2020). Nietzschean meditations: Untimely thoughts at the dawn of the transhuman era.

[25] Jonas Hans (1992). The burden and blessing of mortality. Hastings Center Report.

[26] Kass, Leon R. 2001. L’chaim and its limits: Why not immortality? *First Things*, May, 11pp. https://faculty.utrgv.edu/jose.martinez/General/GenBioCloning.pdf. Accessed May 18 2023. 11933947

[27] Kass Leon R (1985). Toward a more natural science: Biology and human affairs.

[28] Heidegger, Martin. 1962. *Being and time*. Trans. John Macquarrie and Edward Robinson. New York: Harper and Row.

[29] Haugeland, John. 2000. Truth and finitude: Heidegger’s transcendental existentialism. In *Heidegger, authenticity, and modernity: Essays in honor of Hubert L. Dreyfus*, Vol. 1, ed. Mark A. Wrathall and Jeff Malpas, 43–77. Cambridge: MIT Press.

[30] Plato. 1997. *Symposium*. In *Plato: Complete works*, ed. John M. Cooper, 458–505. Indianapolis: Hackett.

[31] Guest Paul C, Guest Paul C (2019). Of mice, whales, jellyfish and men: In pursuit of increased longevity. Reviews on biomarker studies in aging and anti-aging research.

[32] Kant, Immanuel. 1998. *Critique of pure reason*. Trans. and ed. Paul Guyer and Allen W. Wood. New York: Cambridge University Press.

[33] Williams, Garrath. 2023. Kant’s account of reason. *Stanford Encyclopedia of Philosophy*. https://plato.stanford.edu/entries/kant-reason/. Accessed July 2 2023.

[34] de Grey Aubrey, with Michael Rae. (2007). Ending aging: The rejuvenation breakthroughs that could reverse human aging in our lifetime.

[35] Dauphin V. Barry, Abell Steven (2010). Infinite adolescence: A psychoanalytic exploration of tantalizing promises inherent to the singularity. Psychoanalytic Review.

[36] Guyer Paul (2014). Kant.

[37] Wolff Robert Paul (1963). Kant’s theory of mental activity: A commentary on the transcendental analytic of the “Critique of pure reason”.

[38] Humphrey Ted B (1973). The historical and conceptual relations between Kant’s metaphysics of space and philosophy of geometry. Journal of the History of Philosophy.

[39] Höffe Otfried (2009). Kant’s “Critique of pure reason”: The foundation of modern philosophy.

[40] Kant, Immanuel. 2015. *Critique of practical reason*. Rev. ed. Trans. and ed. Mary Gregor. Cambridge: Cambridge University Press.

[41] Zaleski Carol, Post Stephen G, Binstock Robert H (2004). In defense of immortality. The fountain of youth: Cultural, scientific, and ethical perspectives on a biomedical goal.

[42] Aquinas, Saint Thomas. 2012. *Summa theologiae*. Prima pars, 1–49. Trans. Father Laurence Shapcote. Ed. John Mortensen and Enrique Alarcón. Lander, Wyoming: Aquinas Institute for the Study of Sacred Doctrine.

[43] Bostrom Nick, Gordijn Bert, Chadwick Ruth (2008). Why I want to be a posthuman when I grow up. Medical enhancement and posthumanity.

[44] Oxford English Dictionary. 2023. Oxford: Oxford University Press. https://www.oed.com/. Accessed August 30 2023.

[45] Church, Jeffrey. 2022. Kant on death and the purpose of human life. In *Political theory on death and dying*, ed. Erin A. Dolgoy, Kimberly Hurd Hale, and Bruce Peabody, 310–319. New York: Routledge.

[46] Hobbes, Thomas. 2006. *Leviathan*. Ed. Karl Schuhmann and G. A. J. Rogers. London: Bloomsbury.

[47] Kant, Immanuel. 1997. *Lectures on ethics*. Ed. Peter Heath and J. B. Schneewind. Trans. Peter Heath. Cambridge: Cambridge University Press.

[48] Kant, Immanuel. 1983. *Perpetual peace and other essays on politics, history, and morals*. Trans. Ted Humphrey. Indianapolis: Hackett.

[49] Gurofsky Simon R (2020). On the putative possibility of non-spatio-temporal forms of sensibility in Kant. European Journal of Philosophy.

[50] Aristotle. 1988. *Physica*. Ed. W. D. Ross. Oxford: Clarendon Press.

[51] Kurzweil, Ray, and Terry Grossman. 2004. *Fantastic voyage: Live long enough to live forever*. Rodale. 19745481

[52] Feinberg, Joel. 1978. Voluntary euthanasia and the inalienable right to life. *Philosophy and Public Affairs* 7(2): 93–123. https://www.jstor.org/stable/2264987. 11661543

[53] Stone Sophia A, Chatterjee Deen K (2011). Harm principle. Encyclopedia of global justice.

[54] Lyreskog, David M., and Alex McKeown. 2022. On the (non-)rationality of human enhancement and transhumanism. *Science and Engineering Ethics* 28, Article 52: 18pp. 10.1007/s11948-022-00410-4. 10.1007/s11948-022-00410-4 PMC9626409 36318337

[55] Cunningham Anthony (2001). The heart of what matters: The role for literature in moral philosophy.

[56] Nussbaum Martha C (1995). Poetic justice: The literary imagination and public life.

[57] Gorman AG (2016). Williams and the desirability of body-bound immortality revisited. European Journal of Philosophy.

[58] Buben Adam (2022). Do immortals need an eject button? Sartre and the importance of always having an exit. European Journal of Philosophy.

[59] Schramme Thomas (2013). “I hope that I get old before I die”: Ageing and the concept of disease. Theoretical Medicine and Bioethics.

[60] Levin Susan B (2019). Why organ conscription should be off the table: Extrapolation from Heidegger’s *Being and time*. Sophia.

[61] Nuland Sherwin B (1994). How we die: Reflections on life’s final chapter.

[62] Gillman Neil, Post Stephen G, Binstock Robert H (2004). A Jewish theology of death and the afterlife. The fountain of youth: Cultural, scientific, and ethical perspectives on a biomedical goal.

[63] Gadamer, Hans-Georg. 1972. *Wahrheit und Methode*. 3rd exp. ed. Tübingen: J. C. B. Mohr.

[64] North Michael S, Fiske Susan T (2015). Modern attitudes toward older adults in the aging world: A cross-cultural meta-analysis. Psychological Bulletin.

[65] American Academy of Facial Plastic and Reconstructive Surgery, Inc. 2019. News and patient safety. https://www.aafprs.org/AAFPRS/News-Patient-Safety/News.aspx. Accessed December 13 2022.

[66] Brennan Cian (2023). Weak transhumanism: Moderate enhancement as a non-radical path to radical enhancement. Theoretical Medicine and Bioethics.

